# Food Innovation in the Frame of Circular Economy by Designing Ultra-Processed Foods Optimized for Sustainable Nutrition

**DOI:** 10.3389/fnut.2022.886220

**Published:** 2022-05-03

**Authors:** Francesco Capozzi

**Affiliations:** ^1^Interdepartmental Centre for Industrial Agrofood Research—CIRI Agrofood, Alma Mater Studiorum-University of Bologna, Cesena, Italy; ^2^Department of Agricultural and Food Sciences (DISTAL), Alma Mater Studiorum-University of Bologna, Cesena, Italy

**Keywords:** by-products, food processing, food-omics, functional food, sustainability

## Abstract

Despite the large debate about the relationship between ultra-processed foods and the prevalence of some diet-related diseases, the innovative potential of various processing technologies has been evidenced in pathways that could lead to modifications of the food matrix with beneficial health effects. Many efforts have been directed toward the conjugation of a healthy diet and sustainable exploitation of natural resources for the preparation of accessible foods. This minireview highlights the possible links between processing, sustainability, and circular economy through the valorization of by-products that could be exploited to prepare nutrient-rich ingredients at lower economic and environmental costs. The assessment of the quality and safety of functional foods based on ingredients derived from food waste requires a more robust validation by means of the food-omics approach, which considers not only the composition of the final products but also the structural characterization of the matrix, as the bioaccessibility and the bioavailability of nutrients are strictly dependent on the functional characteristics of the innovative ingredients.

## Introduction

Six classification systems have been identified to classify foods and beverages based on processing levels, including the International Food Information Council, the International Agency for Research on Cancer, and NOVA classifications (1). In particular, the NOVA classification system introduced the term “ultra-processed” food (UPF) as a new class that adds to the previous triad of unprocessed (NPF), minimally processed (MPF), and processed foods (PF). As processing changes the physical, chemical, and biological properties of foods, the level (intensity, duration, and number of processes) and type of technology used in the processing operations are relevant for determining shelf life, food safety and quality, and the bioavailability of nutrients (2). UPFs are formulations of ingredients, most of exclusive industrial use, that result from a series of industrial processes, including the fractioning of whole foods into substances, chemical modifications of these substances, assembly of unmodified and modified food substances, frequent use of cosmetic additives and sophisticated packaging (3). This way, food classification moves away from traditional food groupings (e.g., “grains and grain products” and “meat and meat products”), and not necessarily consider established methods based on nutrients (e.g., sodium, dietary fibers, saturated fat, and added sugars) (4).

As consumers find a wide variety of affordable, palatable, accessible, and stable UPFs, they now account for a substantial share of overall food intake, with significant heterogeneity across countries and socioeconomic strata. For example, the lowest intake was observed in Italy with around 10% of total kcal from UPFs and the highest in the United States and the United Kingdom with over 50% of total kcal from (5). It was recently observed that UPFs consumption is associated with a deterioration in diet quality, with UPFs intake being negatively correlated with fibers and protein and positively correlated with sugar, fat, and saturated fat intake (6). From a nutrient point of view, UPFs have on average a higher energy density (2.3 vs. 1.1 kcal/g) and a lower nutrient density than minimally processed foods (7). Moreover, changes in the food matrix can also alter nutrient bioavailability and UPFs tend to be more hyperglycemic than MPFs (8). Moreover, the high palatability of UPFs has the potential to promote a faster eating rate and energy overconsumption, as they are consumed more quickly, also due to a reduced oro-sensory exposure time, delaying the onset of satiation (9).

Although epidemiologic evidence has been postulated for the association between UPFs and health, most of the observations linking UPFs to diet-related diseases derived from studies conducted with food questionnaires that are not specifically validated for UPFs, although some efforts have been made in this direction [e.g., (10, 11)]. Descriptions of UPFs within the NOVA system vary with distinguishing features including single vs. 2–3 vs. ≥5 more ingredients, natural/fresh vs. imitation or industrial, and whole foods vs. fractioned substances. This means that different studies may have classified the same food as UPFs or not based on the distinguishing feature used for classifying foods (5). Even for the only intervention study so far conducted to investigate the impact of UPF on human health, the choice of UPFs was arbitrary and not representative of the whole range of available items, notably spanning different nutrient/energy densities (12). Thus, further efforts are essential to confirm the results previously obtained and to investigate further the association between UPFs consumption and health status, also considering the actual contribution within different dietary patterns, which has been less investigated to date (5). Moreover, the series of inconsistencies originated from the lack of a shared definition of food categories associated with UPFs, and a common language among nutritionists, food scientists, and technologists, have led to a semantic debate, rather than a scientific one, on the motivations for adopting strategies that reduce the consumption of UPFs, as a whole (13). Indeed, the drastic reduction or elimination of the availability of all categories of UPFs without simultaneous consideration and efforts to replace them with better, affordable, and practical alternatives is not a winning strategy. Nevertheless, eliminating UPFs that provide many desirable properties (economic, microbiological safety, nutrient fortification, extended shelf life, and affordability) can only worsen existing disparities in food insecurity (14). Moreover, ingredients and processing should not be considered independently, as the nutritional content largely depends on the recipe (ingredients) and not exclusively on the preparation procedure. Avoiding foods considered UPFs, such as whole/enriched bread and grains or flavored milk, may not address obesity but may reduce the intake of folate, calcium, and dietary fibers (15).

Rather than eliminating ready-to-eat (RTE) or ready-to-heat (RTH) UPFs, their usefulness in food use and the household should be recognized, considering that their reformulation, rather than elimination, could have a more significant impact on improving nutritional quality and health at the population level (14). For this purpose, some nutrient-based quality descriptors should be selected, such as the nutrient-rich food index (NRFI) based on the content of protein, fibers, vitamins, minerals, saturated fat, and added sugar and sodium (16). Studies comparing UPFs with respect to their NRFI show that most UPFs have low nutritional quality, but some are high, thus evidencing that the inclusion of an item in the category of UPFs is not a synonym for bad quality. According to the same index, most MPFs have high nutritional quality, but some are still low quality, and the consumption of the latter should be minimized as well (7). Taking for granted that the simple NOVA classification cannot be adopted alone, and a better definition of food quality must include the actual molecular composition, new formulations for UPFs could be achieved, improving their nutritional quality.

## Adding Nutritional Value to Ultra-Processed Foods

**FIGURE 1:**
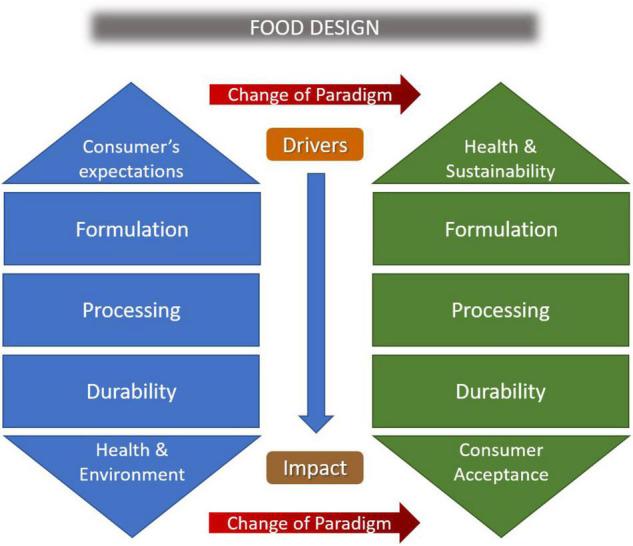
A new paradigm in the development of innovative products should be considered by the industrial food value chain, where the healthiness is no more a tentative consequence of the intended result. Current efforts are mainly directed to fulfill the consumers’ compliance, based on tangible attributes like sensorial acceptance and economic affordability. In a new food system, the main driver for food design must be the healthiness attribute, while selecting sustainable ingredients and technologies, to get the best market acceptance.

While the dilemma on the link between ultra-processing and the onset of food-related diseases cannot yet be definitively resolved, the idea that the composition of UPFs can be improved through reformulations richer in noble nutrients remains valid. Thus, reformulation strategies tend to enrich largely consumed foods with proteins, vitamins, or dietary fibers, while reducing, if not eliminating, nutrients that in prevalent diets normally exceed the recommended doses, such as saturated fats, sodium chloride, or simple sugars. Such a strategy is driving the development of “Healthy” UPFs, often plant-based alternatives, carrying nutrition claims such as “fat-free,” “reduced salt,” “low sugar” or “added fibers” according to the nutritional guidelines and the CE Regulation n. 1924/2006. Other “healthy” UPFs, such as fortified bread, have been suggested to be important sources of vitamins and minerals, and the avoidance of such UPFs may lead to micronutrient deficiencies (17). It is worth noting that foods are not simple homogeneous mixtures and formulations must also consider the nature of the substituting ingredients, as the structure of the food matrix, and consequently the bioavailability of the nutrients, is deeply affected by the tight coupling of chosen sources and applied technologies. To this end, food technologists, inspired by nutritional goals, can take advantage of ultra-processing technology to design healthier foods, making them more appreciated, therefore more frequently consumed (Figure 1). While food fortification would be a solution, any strategy based on it would have to consider the actual exposures of different population groups. Conversely, the transition to the reduced content of some ingredients, e.g., salts or other additives, cannot be pursued by simply eliminating them, so as not to expose consumers to pathogenic risks. Thus, reformulating does not mean a simple elimination or substitution but can involve a complete redesign of the food in its entirety. In this regard, the interconnection between different types of experts is mandatory to promote solid food design for healthy and necessary products tailored to the right target group.

In addition to the mandatory safety requirement, another factor must be considered when designing a tailor-made food, namely consumer acceptability. Even if the elimination of an ingredient does not involve safety risks, the very existence of a product in the form that consumers do expect can be compromised. In bakery products, such as cookies and cakes, sugar is one of the main components, contributing up to 30–40% of the total recipe, and intense scientific research is performed regarding the replacement of sugars with more healthy alternatives. However, the reformulation of confectionery and bakery products with a substantial reduction in sugars has proven difficult due to the multiple functionalities that sugars exert in bakery products, next from simply providing sweetness. Sucrose cannot just be replaced by a single compound, but a mixture of compounds, assuming that sweetness and color can be decoupled from structural and textural attributes like firmness, crispiness, and dryness. Rather than proceeding empirically, by trial and error, to find an optimal formulation, considerable progress has been made in building digital tools to predict the outcome of a substitution. Recently, van der Sman and Renzetti (18) made much progress in developing a numerical model, incorporating predictive thermodynamic theories, for optimization of sugar-reduced formulations in an effective and efficient manner, also providing guidance for replacement strategies (e.g., by the inclusion of dietary fibers). The technological operations underlying the production of UPFs are often accused of being destructuring the natural matrix that constitutes food, a fact considered deleterious for the quality of food, as the matrix is seen as an intrinsically positive constituent of the raw food, which is lost in its transformation into edible food. It has been suggested that the presence of acellular nutrients, food additives, non-energetic artificial sweeteners, and possibly advanced glycation end products, as well as the lack of fermentable fibers and phytochemicals, could be responsible for the altered composition and metabolism of gut microbes (19).

As mentioned before, the destruction of the barriers exerted by the compartmentalization of animal tissues and, even more, of plant tissues, makes nutrients immediately available for absorption, creating glucose peaks in hematic concentrations that can overload normal physiological functions, thus generating a low-grade systemic inflammation (20). For this reason, considering the disintegrating capacity, the most offending practices for food processing include the fractionation, which transforms raw ingredients or whole foods into simple molecules such as sugars, oils and fats, proteins, starches, and fibers. Some of these substances are then subjected to hydrolysis hydrogenation, or other chemical modifications to make them even more suitable for their incorporation in formulations, using industrial techniques such as extrusion or molding. It is worth mentioning here that some population groups (e.g., the elderly, infants, or pregnant women) can benefit from readily available crucial nutrients. Likewise, nutrient deficiency associated with poor bioavailability is also relevant for malnourished populations who consume only raw or whole foods. Certainly, it is not only the number of nutrients that counts but also, and above all their quality. Therefore, it is necessary to ensure that the nutrients made available best meet nutritional needs.

A further criticism of the artificial nature of the UPFs assembly is the use of cosmetic additives, which include substances selected to improve aroma, enhance flavor, give attractive color, stabilize emulsions or gels, sweeten, thicken or, conversely, increase the volume of food preparation. These classes of additives can also be used to hide undesirable sensory properties created by ingredients or processes. For this reason, their use is seen as a camouflage of poor quality, and consumers are pushing for “clean label” food, i.e., with low or no artificial additives while attributing a nutritional detriment to their use.

## The Consumer’s Choice for “Clean Label” Foods and New Natural Improvers for Ultra-Processed Foods

The “clean label” choice, initially associated with the absence of E-numbers in the list of ingredients, has driven the food industry to communicate whether a certain artificial ingredient or additive is not present in the food product (21). Additives are not optional as they are necessary for food stability and consumer acceptance. Even more so, when some excess ingredients are replaced by others, e.g., in “vegetable meat” or “gluten-free pasta,” additives are mandatory. The challenge is to find natural ingredients instead of refined artificial ones, with optimal properties and suitable for use in food processing. Therefore, replacing a list of additives with a few natural ingredients that collect all the properties necessary to stabilize foods, would move UPFs away from the term “ultra” and wash them to be “clean.” Thus, there is a significant trend toward the consumption of products with a clean label which is driving food researchers and technologists to explore new “natural” ingredients and processes for the preparation of novel foods. This new way of conceiving healthy and “green” foods must be demonstrated by scientific evidence with a measure of impacts on the human metabolome, looking at the food topic with a new perspective offered by the “foodomics approach” (22).

The food industry is constantly looking for new ingredients able to replace sugar’s technological functionality while satisfying the consumer’s request for a clean label. For instance, based on corn (*Zea mays*) and chickpeas (*Cicer arietinum*), a fiber syrup was tested as a bulking agent in cookies to reach up to 50% sugar reduction, allowing to obtain sugar-reduced cookies qualified for “reduced in sugar” and “high in fiber” nutritional claims (23). Regarding grain-based foods, it is noted that the world consumption of whole grains and legumes is significantly below what is recommended. The focus is being placed on new raw materials from little-used ancient cereals, pseudo-cereals, or legumes, but the main challenge remains to improve their technological and sensorial properties while avoiding the use of additives that would deviate them from the clean label. For this reason, non-thermal technologies have been explored as an alternative to additives (24).

As most additives are used to stabilize foods, more and more solutions are sought to replace them with treatments that adequately modify the structure of the ingredients to make them stable naturally. Among these, non-thermal treatments are emerging, such as high hydrostatic pressure (HPP), cold gas plasma, ultrasound, ozonation, ultraviolet and pulsed light, aimed at stabilizing food avoiding the use of chemical additives. They were originally developed to inactivate microorganisms and enzymes in foods, in alternative to conventional processing methods (e.g., boiling and steaming) that destroy nutritional components. Several studies have shown that such novel processing techniques generally perform better in maintaining the original characteristics of foods (25). In the case of cereal products, these technologies can be used at low temperatures to modify the most important component of wheat flour, i.e., gluten and starch, which are responsible for the rheological properties of wheat flour dough. Non-thermal technologies can be responsible for the denaturation of gluten or the debranching of starch. These changes can result in increased numbers of protein aggregates that can directly affect the elasticity and strength of the dough. Studies have shown that HHP can produce partially gelatinized starch that can improve water retention and rheological characteristics of the dough. In bread making, the damaged-starch content is important for dough hydration; however, the damaged-starch content greater than 10% and the presence of pre-gelatinized starch can overhydrate the flour and make it sticky, making the dough difficult to handle (24). For this reason, in the development of each specific formulation, the appropriate parameters should be finely optimized for each ingredient-technology pair. At the same time, the impact on the bioaccessibility and bioavailability of the nutrients together with the sensorial properties of the new final product should be evaluated.

By combining new sources of nutrients and natural additives with the use of improving technologies, to make these ingredients more effective in their function, the exploitation of food industry by-products is an almost automatic consequence. This strategy, called circular economy, is successful when new nutritional sources, of equal or better quality than those used conventionally, are exploited because it has the merit of linking the demand for healthier foods with the sustainability of their production. Still remaining in the field of cereal-based products, the use of bread’ leftovers that are withdrawn from the market due to texture issues, but that has not experienced microbial deterioration, is being considered. In this regard, research has shown that these materials could be incorporated to produce bread, cakes, or cookies (26). However, these additions can have negative implications on the quality of final products, especially on their organoleptic acceptability, which makes necessary focused research to minimize these negative problems, mainly by optimizing the percentages that can be incorporated to achieve final products with acceptable organoleptic properties.

**FIGURE 2:**
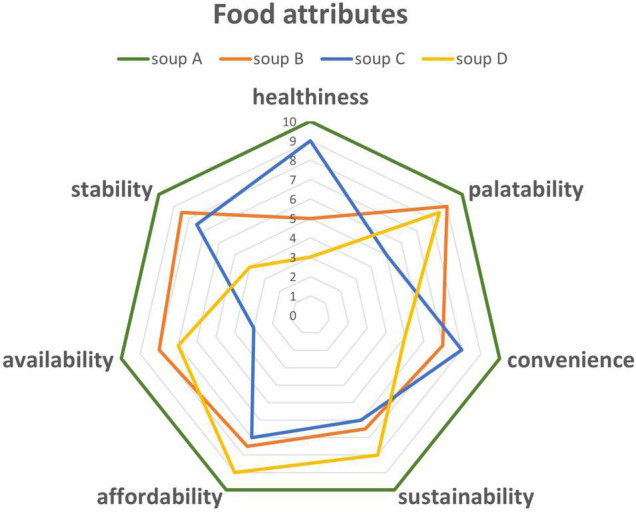
The spider plot allows the consumer a rapid appreciation of all the attributes of food quality. Each colored line represents a food with different quality attributes. For instance, soup A is “healthier” than soup C, which conversely is more appreciated for its sustainability. The main challenge in this kind of representation is the arbitrary rationale behind the assignment of relative scores to each attribute, that must be normalized in an appropriate ponderal scale so that healthiness has the proper weight compared to the other quality attributes.

Thus, the incorporation of ingredients derived from valorized agro-industrial by-products in “clean label” foods may be seen as a possible solution for replacing “artificial foods” with natural recipes. Interestingly, not only by-products obtained during the processing of cereals, such as bran and germs, are incorporated in grain-based food but also by-products from the fruit and vegetable industry (27). Martins et al. (28) collected, in a comprehensive review, a large collection of data on fruit by-products, including not only their content of nutrients and bioactive compounds but also their respective rheological and functional properties, in the optic of possible exploitation as natural ingredients in the bakery industry, with stabilizing and enhancing sensorial properties. However, using whole-wheat flour or the addition of dietary fibers, seeds, fruits, or flours from different sources is not a trivial practice, as they disrupt the starch–gluten matrix by affecting the viscoelasticity of the dough, resulting in lower-quality bread (24). The processing technologies, in particular those with a lower impact on the nutrient content, are increasingly explored to conjugate the content of sustainable and minimally fractionated ingredients with good taste. The great challenge is to collect ingredients, containing healthy nutrients that are deficient in a large part of the population, valorizing by-products and leftovers to exert a lower impact on the natural resources. The final goal of such a valorization route is a comprehensive scoring system that summarizes the quality of the food product in a way that sustainability and healthiness should be correctly emphasized together with the other quality attributes conventionally addressed by the food industry to meet the consumers’ acceptance (Figure 2).

## Ingredients From Waste to Food

In the previous sections, it has been highlighted how important, and at the same time difficult, is to design new foods using new natural sources to obtain ingredients and applying technologies capable of making these new ingredients suitable for the production of palatable food. The next step, addressed in this section, is to make this process also sustainable from the point of view of the impacts on natural resources. This need has become an absolute priority on the agendas of all policymakers. In fact, the EU prioritizes the sustainability of food systems and circular economy to reduce the environmental impact. It recommends the reuse of all waste suitable for human consumption to be reintroduced into the food chain (29).

The circular bioeconomy strategy starts from the premise that 30% of the world’s food production is wasted and the whole current agrifood system consumes about 70% of the world’s freshwater. Thus, recovering ingredients from wastes means a partial recovery of water, as well as other natural resources, for purposes, i.e., the human feeding, that was originally intended as the target destination of such exploitation. The adoption of advanced technologies, such as biotechnology, can procure novel foods and feed ingredients, to privilege pathways where functional properties are safeguarded and directly valorized into ingredients. A very recent review by Javourez et al. (30) classified 150 different biomass residues from ten categories of raw materials: (i) wood-related, (ii) primary crop, (iii) manure, (iv) food waste (from households or the service sector such as restaurants, etc.), (v) sludge and wastewater, (vi) green residual biomass, (vii) slaughterhouse by-products, (viii) agrifood co-products, (ix) C1 gases, and (x) others. They were further logically analyzed according to four ideal building blocks for conversion pathways transforming waste into ingredients for food or animal feed:

(i) Enhancement, i.e., increase of quantity, preservation, or accessibility of nutrients, without removal of any components.
(ii) Cracking, i.e., deconstruction of extremely recalcitrant structures to facilitate the release of nutritional compounds.
(iii) Extraction, i.e., selective solubilization and/or separation of a target fraction from a matrix
(iv) Bioconversion, i.e., conversion of feedstocks into nutritional ingredients using the metabolic processes of living organisms.

A resource is considered a food or feed grade ingredient when the nutrients contained within the ingested ingredients are released and assimilated without adverse effects. Besides composition and structural characteristics, safety is determined by the inherent features of the digestive tract. For this reason, what is considered safe for ruminants, could not be the same for humans. The nutritional quality of an ingredient is characterized by three main factors: (i) the absence of anti-nutritional compounds, (ii) the degree of structural complexity, and (iii) the concentration of macro- and micronutrients. Most residual biomasses cannot be considered food grade. Anti-nutritional factors often arise because of large heterogeneity and/or biological activity found in most of the leftovers (e.g., in food waste). Moreover, they include recalcitrant matrices that are incompatible with direct edibility. Accordingly, to transform waste into ingredients, a sequence of operations is required that breakdown structural barriers, remove harmful compounds, and possibly, enriches the assimilable nutrients.

One example of enhancement is represented by whole apple pomace (containing pulp, peel, seeds, and stems) collected from juice factories, immediately after pressing. Then, they are dehydrated at the industrial level and ground into flour used to fortify cookies, enriched with dietary fibers and flavonoids, produced by replacing 25% of wheat flour (31). Also, brewers’ spent grains milled into bakery flour is another example of an enhancement pathway: the bitter taste and unpleasant mouthfeel, is eliminated by cleaning, drying, and milling the spent grains, and the ingredient is used to enrich dry pasta (up to 135% fiber, 57% resistant starch, 85% β-glucan), involving minimal effects on sensory properties of cooked pasta (32).

As far as the extraction pathway is concerned, proteins from defatted rice bran represent a valuable nutrient that is easily recovered by alkaline extraction, to obtain concentrate ingredients useful for preparing protein-enriched flour substitute in biscuits. A two-fold increase in the protein content of biscuits has been reported with the incorporation of 15% rice bran protein concentrate (33). Grass and vegetable leaves were also exploited as a source of a beneficial protein isolate that resulted in good gluten substitution in the bakery. A sequence of industrial operations, including mechanical disruption to extract green juice, removal of chloroplast membrane through heat treatment coagulation, ultrafiltration, hydrophobic column adsorption, and spray drying, was optimized to extract the rubisco protein from the cellulosic structure rich in phenol compounds (34).

From lignocellulosic feedstocks, by means of a cracking pathway, a detoxified hemicellulose hydrolyzate, has been manufactured. The hemicellulose hydrolyzate has been obtained through dilute acid hydrolysis and further bio-converted by fermentation with *Candida athensensis* to produce xylitol as an alternative sweetener (35). Feather is another interesting source of free amino acids and functional short-chain peptides, obtained by enzymatic conversion of keratin (yield of 50-60%). Although keratin constitutes <80% of dry matter of feathers, its resistance to digestibility makes such a protein useless as a nutrient (36).

The last pathway for the transformation of wastes in food ingredients is bioconversion: insects farming on food waste and microalgae cultured on aquaculture wastewater are emerging as sustainable practices for the exploitation of sources, coupling very high conversion efficiency of natural resources with low environmental impact and high nutrient density. Fractionation of insect biomass is currently the best option for broader adoption in western countries, as consumers may be reluctant to accept whole insects for cultural reasons. This suggests that the preferable approach would be to transform insects in meals, using protein and other fractions as food/feed ingredients. Processing of larvae into separate fractions may also address microbiological safety issues by killing bacteria during drying and extraction steps (37).

## Conclusion

The definition of UPF for nutritional assessment is controversial, as it is mainly based, according to the NOVA classification, on the number of ingredients, which are refined and mostly artificial additives. However, since foods are not simple homogeneous mixtures, additives are not optional to obtain products that are also acceptable to consumers and appreciation is one of the most important drivers for consumption. Artificial additives are currently used to achieve the stability and good sensory attribute of foods, but these ingredients are viewed negatively by UPF detractors, describing these chemicals as man-made, non-natural, and possibly harmful to human health. For this reason, the reduction or elimination of these artificial additives is considered a further improvement also by nutritionists. Replacing a list of additives with a few structured ingredients that collect all the properties necessary to stabilize food, would move UPFs away from the term “ultra” and make them “clean label”. The challenge is to find optimal natural ingredients instead of refined artificial ones, satisfying another emerging need dictated by society: zero impact on the environment.

The ecological transition, defined as the “Green Deal” by the European Union, must be applied in every production sector, especially in the agri-food sector where great exploitation of natural resources takes place. Greater efficiency of the production system, through more precise agronomic and breeding practices, is certainly the main pathway. However, considering that one-third of food production becomes waste, it is necessary to develop strategies that minimize waste, for example by increasing the stability of products over time. In addition, it must also recover value from any form of a by-product that contains non-negligible quantities of nutrients. Food technologies can help exploit ingredients derived from by-products through inclusion in foods with high functional and sensory qualities. However, this use of alternative ingredients and innovative technologies cannot ignore the healthiness of the final product. For this reason, the ever-closer collaboration between food technologists and nutritionists is necessary for the development of foods that will be part of the future diet in the name of environmental sustainability.

## Author Contributions

FC: accountable for the whole content of the work.

## Conflict of Interest

The author declares that the research was conducted in the absence of any commercial or financial relationships that could be construed as a potential conflict of interest.

## Publisher’s Note

All claims expressed in this article are solely those of the authors and do not necessarily represent those of their affiliated organizations, or those of the publisher, the editors and the reviewers. Any product that may be evaluated in this article, or claim that may be made by its manufacturer, is not guaranteed or endorsed by the publisher.
